# Supplementary material for: Circular RNA- and microRNA-Mediated Post-Transcriptional Regulation of Preadipocyte Differentiation in Adipogenesis: From Expression Profiling to Signaling Pathway
Source: Int J Mol Sci. 2023 Feb 25;24(5):4549. doi: 10.3390/ijms24054549 (PMC10002489; doi:10.3390/ijms24054549)
Supplement: Supplementary file 1 [file ijms-24-04549-s001.zip › ijms-2180604-supplementary.pdf]

Suppl. Table S1. Expression of circRNAs in WAT and BAT of the human and animals on preadipocyte differentiation (based on reference [51])

No.	Host gene	Mouse WAT-1[a]	Mouse WAT-2	Human WAT	Yak WAT	Mouse BAT
	Zhang PP et al. (2021) [51]		Arcinas et al. (2019) [49]		Zhang Y et al., (2020) [53]	Zhang P et al. (2021) [52]
**(A) Up-regulated circRNAs on differentiation (from high to low)[b]**						
1	Acss3	mmu_circ_Acss3_1	3+	+	+	4+
2	NA	chr17:34872348-34952518	-	-	-	+
3	Acss3	mmu_circ_Acss3_2	3+	+	+	4+
4	Mlxipl	mmu_ circ_Mlxipl	+	-	-	+
5	NA	chr17:34877211-34956589	-	-	-	-
6	Pcsk5	mmu_circ_0000937 (mmu_circ_Pcsk5)	+	+	2+	+
7	Bmper	mmu_circ_0015472_1 (mmu_ circ_Bmper)	-	+	-	+
8	Nfia	mmu_circ_Nfia	+	+	-	+
9	Cacna1d	mmu_circ_0005172 (mmu_circ_Cacna1d)	-	-	-	-
10	Fkbp5	mmu_circ_Fkbp5	-	-	8+	+
11	Bmper	mmu_circ_0015472_2 (mmu_ circ_Bmper)	-	+	-	+
12	Selenbp1	mmu_circ_Selenbp1	+	-	-	+
13	Zbtb16	mmu_circ_Zbtb16	3+	+	2+	2+
14	Ror1	mmu_circ_Ror1	-	+	2+	+
15	Egfr	mmu_circ_0002861 (mmu_circ_Egfr)	+	-	-	+
16	Tgfbr2	mmu_circ_0001853 (mmu_circ_Tgfbr2)	+	3+	2+	+
17	Cped1	mmu_circ_0001447 (mmu_circ_Cped1)	-	+	7+	+
18	Fndc3b	mmu_circ_0010609 (mmu_circ_Fndc3b)	2+	9+	5+	+
19	Slc10a7	mmu_circ_Slc10a7	3+	-	-	+
20	Gbe1	mmu_circ_0006376 (mmu_circ_Gbe1)	3+	3+	-	+
21	Pcsk5	mmu_circ_0000938 (mmu_circ_Pcsk5)	+	+	2+	+
22	Bckdhb	mmu_circ_0001822 (mmu_circ_Bckdhb)	7+	-	-	2+

23	Arhgap10	mmu_circ_Arhgap10_1	10+	9+	2+	+
24	Arhgap10	mmu_circ_Arhgap10_2	10+	9+	2+	+
25	Fancl	mmu_circ_0000248 (mmu_circ_Fancl)	+	2+	-	-
26	Acvr2a	mmu_circ_0001017 (mmu_circ_Acvr2a)	+	+	2+	+
27	Med13l	mmu_circ_0001396 (mmu_circ_Med13l)	2+	2+	2+	+
28	Marchf3	mmu_circ_0007399 (mmu_circ_Marchf3)	+	-	-	+
**(B) Down-regulated circRNA during adipogenesis (from high to low)[b]**						
1	Pdlim5	mmu_circ_Pdlim5	+	6+	6+	+
2	Slc7a11	mmu_circ_0010676 (mmu_circ_Slc7a11)	-	-	+	+
3	Rad18	mmu_circ_0001504 (mmu_circ_Rad18)	-	+	3+	-
4	Megf8	mmu_circ_0014097 (mmu_circ_Megf8)	-	-	-	-
5	Trpc6	mmu_circ_0001743 (mmu_circ_Trpc6)	-	-	-	-
6	Cacna1c	mmu_circ_0001511 (mmu_circ_Cacna1c)	-	-	-	3+
7	Nsd2	mmu_circ_0001335 (mmu_circ_Nsd2)	-	-	-	+
8	Zfp532	mmu_circ_0000884 (mmu_circ_Zfp532)	2+	-	-	-
9	Dcbld2	mmu_circ_0000693 (mmu_circ_Dcbld2)	+	+	4+	-
10	Tulp4	mmu_circ_0000723 (mmu_circ_Tulp4)	4+	-	-	+
11	Zbtb38	mmu_circ_Zbtb38	-	-	-	+
12	Pde4d	mmu_circ_0004451 (mmu_circ_Pde4d)	-	+	-	2+
13	Zfx	mmu_circ_0016408 (mmu_circ_Zfx)	+	+	+	-

[a]CircRNAs in host-name nomenclature are provided in brackets below the circBase ID whenever available; NA, not available. [b]The circRNAs are in the order of decreasing expression levels based on the mouse WAT-1 dataset of Zhang PP et al. 2021 [51]. Note that circRNAs Acss3 (rows 1 & 3), Pcsk5 (6 & 21), Bmper (7 & 11) and Arhgap10 (23 & 24) each shows two different isoforms in the dataset. The number of isoforms in the circRNA datasets reported in other studies are shown by the number of "+"; "-" indicates not found in the dataset.

Suppl. Table S2. Molecular features of the selected adipogenesis-associated circRNAs

Host gene	Species	circRNA	circBase ID	Exons	Size (nt)	Best transcript	Chromosomal location
PPARγ	Cattle	bta_circPparγ	bta-circ-0010660	3-5	509	NM_181024	Chr22: 56710574-56775806
FLT1	Cattle	bta_circFlt1	bta_circ_002673	2-3	327	XM_027557508	Chr12: 31656945-31657867
FUT10	Cattle	bta_circFut10	NA	2	295	NM_182987	Chr27: 16348184-16348478
SAMD4A	Human	hsa_circSAMD4A	hsa_circ_0004846	3	519	NM_015589	Chr14: 55168779-55169298

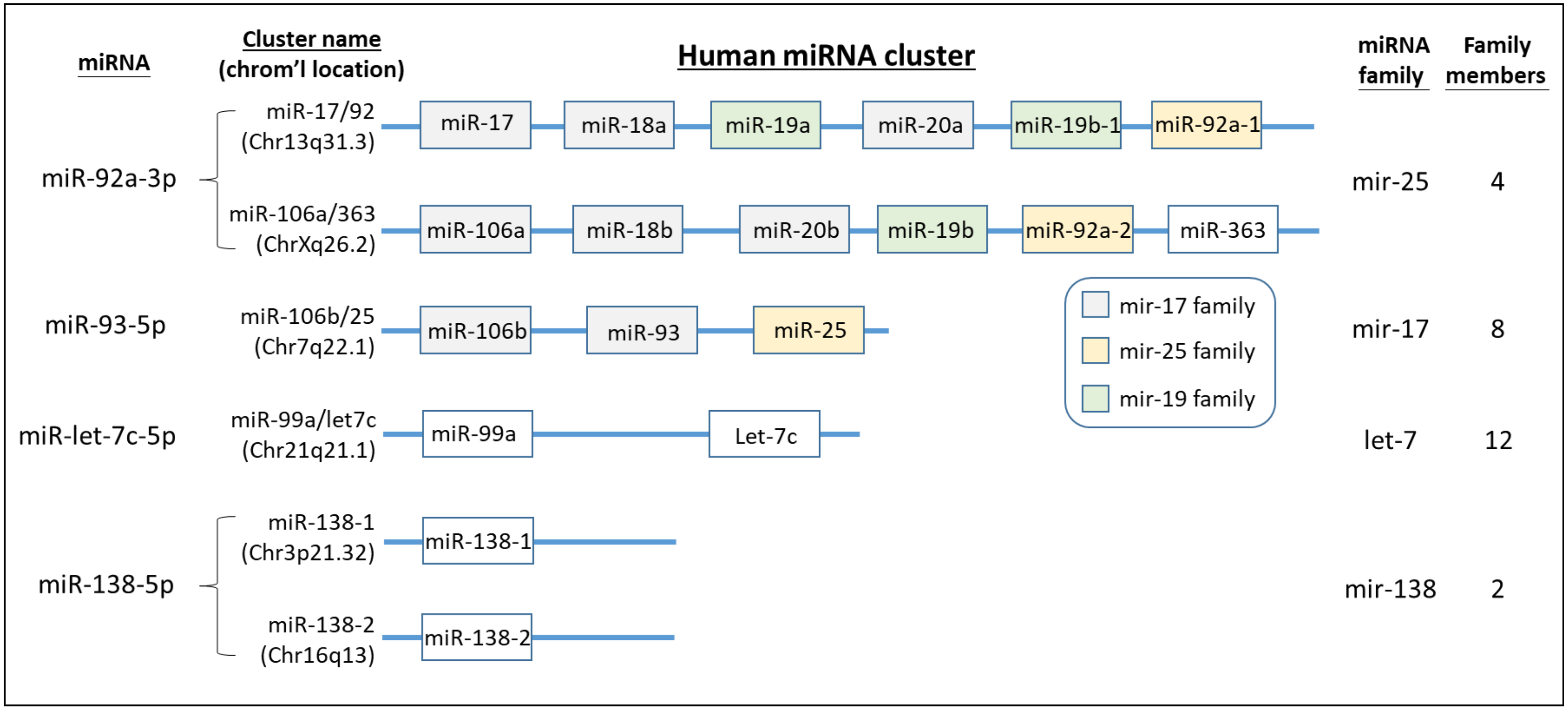

Suppl. Figure S1. MiRNA families and clusters.
